# Marginal zone SIGN-R1^+^ macrophages are essential for the maturation of germinal center B cells in the spleen

**DOI:** 10.1073/pnas.1921673117

**Published:** 2020-05-18

**Authors:** Gabriela Pirgova, Anne Chauveau, Andrew J. MacLean, Jason G. Cyster, Tal I. Arnon

**Affiliations:** ^a^Kennedy Institute of Rheumatology, University of Oxford, OX3 7FY Oxford, United Kingdom;; ^b^Howard Hughes Medical Institute, University of California, San Francisco, CA 94143;; ^c^Department of Microbiology and Immunology, University of California, San Francisco, CA 94143

**Keywords:** marginal zone macrophages, germinal center B cells, marginal zone B cells

## Abstract

The mechanisms that regulate germinal center (GC) B cell responses in the spleen are not fully understood. Here we use a combination of pharmacologic and genetic approaches to delete SIGN-R1^+^ marginal zone (MZ) macrophages and reveal their specific contribution to the regulation of humoral immunity in the spleen. We find that while SIGN-R1^+^ macrophages were not essential for initial activation of B cells, they were required for maturation of the response and development of GC B cells. These defects could be corrected when follicular helper T (Tfh) cells were induced before macrophage ablation or when Tfh responses were enhanced. Moreover, we show that in the absence of SIGN-R1^+^ macrophages, DCIR2^+^ dendritic cells (DCs), which play a key role in priming Tfh responses, were unable to cluster to the interfollicular regions of the spleen and were instead displaced to the MZ. Restoring SIGN-R1^+^ macrophages to the spleen corrected positioning of DCIR2^+^ DCs and rescued the GC B cell response. Our study reveals a previously unappreciated role for SIGN-R1^+^ macrophages in regulation of the GC reaction and highlights the functional specification of macrophage subsets in the MZ compartment.

## Significance

Germinal centers (GCs) are critical for the generation of memory B cells and high-affinity antibody-producing cells. This process is important for protection from acute infections and for establishment of long-lasting immunity in response to vaccination. The microanatomic organization of distinct niches within lymphoid tissues is fundamental for GC responses, as it provides the basis for coordinated interactions between rare antigen-specific cells and antigen-presenting cells. Here we reveal a role for a specialized resident macrophage subset in maintaining positional regulation of a key antigen-presenting cell type within functional niches in the spleen. Our study demonstrates the importance of this regulation to humoral immunity.

The spleen plays a key role in protection from pathogens that have breached mucosal barriers and entered the systemic blood circulation. Most of the blood that enters the spleen first reaches the marginal zone (MZ), a narrow compartment that surrounds the B cell follicles and separates them from the open blood circulation of the red pulp (1). As such, the MZ compartment represents an important interface where systemically disseminated pathogens and antigens are initially filtered. Within this region, two major subsets of resident macrophages have been identified: MZ macrophages and metallophilic macrophages. While both subsets have been shown to play roles in the regulation of immune responses and peripheral tolerance, the specific contributions of each macrophage population to these processes and the mechanisms involved are not well understood (2–6).

Unlike the metallophilic macrophages that lie underneath the marginal sinuses and express high levels of the sialic acid-binding receptor CD169, MZ macrophages are located outside the follicles in the MZ compartment itself and show low to undetectable CD169 expression. In addition, MZ macrophages can be distinguished from metallophilic macrophages by the expression of the scavenger receptor MARCO and the lectin binding receptor SIGN-R1, which are absent in metallophilic cells (3, 4). Notably, while MZ macrophages are typically referred to as a single population of cells, closer examination shows that they are in fact heterogeneous, and that in addition to cells expressing both SIGN-R1 and MARCO on their surface, the outer rim of the MZ also contains a subset of macrophages that express the MARCO receptor but lacks SIGN-R1. The relationship between these two cell subsets is not well understood, and it is possible that they reflect different activation states of cells with a common origin. However, previous studies have found that these two cell subsets not only are phenotypically distinguishable, but also differ in their developmental requirements and kinetics; while MARCO^+^ SIGN-R1^−^ cells are independent of macrophage colony-stimulating factor (M-CSF) and are the first to populate the MZ, SIGN-R1^+^ MARCO^+^ cells take longer to appear (7), and their development is arrested in the absence of M-CSF (8, 9). Moreover, previous studies have shown that SIGN-R1^+^ MARCO^+^ cells, but not SIGN-R1^−^ MARCO^+^ cells, critically depend on interactions with MZ B cells that occupy the compartment, and that when MZ B cells are displaced, SIGN-R1^+^ MARCO^+^ cells are diminished (10, 11). Taken together, these studies support the notion that the MZ macrophage population contains at least two distinct cell types, and that interactions with MZ B cells can modify the balance between them. However, the functional consequences of this interplay and its impact on the generation of high-affinity antibody responses have not yet been explored.

A hallmark of MZ macrophages is their ability to efficiently trap and display antibody-coated antigens from the blood (10, 12). Immune complexes (ICs) captured by MZ macrophages are subsequently transferred to local MZ B cells, which carry and deposit them on follicular dendritic cells (FDCs) inside the follicles (13, 14). Migration of MZ B cells between the MZ and follicular compartments is necessary for this process and is regulated by the interplay between the chemokine receptor CXCR5, which attracts the cells to the follicles, and Sphingosine-1-Phosphate Receptor 1 (S1PR1), which opposes CXCR5-mediated signals and promotes migration toward the MZ compartment (15). Since antigen presentation by FDCs is important for germinal center (GC) B cell responses, this pathway may help to promote the generation of high-affinity antibodies. However, whether MZ B cell-mediated antigen shuttling promotes GC B cell development remains speculative, and the manner by which interactions between MZ B cells and SIGN-R1^+^ MARCO^+^ macrophages may regulate B cell responses in the spleen is currently unknown.

Here we used a combination of approaches, including the development of a mouse model, to explore the specific role of SIGN-R1^+^ MARCO^+^ macrophages (referred to as “SIGN-R1 macrophages” herein) to the regulation of B cell responses in the spleen. Our findings demonstrate a high level of functional specification between closely related macrophage subsets within the MZ compartment and reveal a critical role for these cells in maintaining intact functional niches. Furthermore, our work highlights the importance of MZ B cell– SIGN-R1 macrophage interactions for adaptive immunity and demonstrates that these interactions play important roles that go beyond contributions to antigen deposition on FDCs. Taken together, these results establish that intact SIGN-R1 macrophage function in the spleen is a critical requirement for a productive humoral immune ;response.

## Results

### MZ B Cells Are Necessary for GC B Cell Responses in the Spleen.

**Fig. 1.**
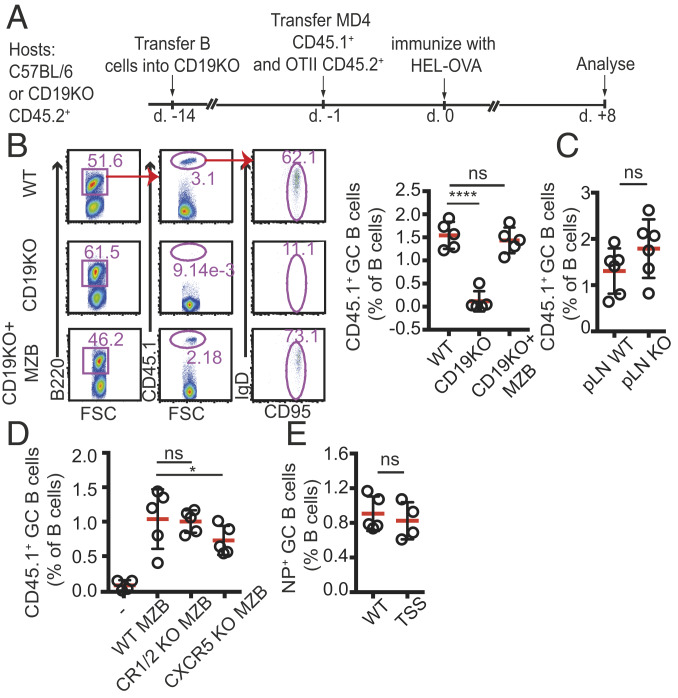
GC B cell responses in the absence of MZ B cells. (*A*) Graphic representation of the experimental protocol. (*B*) Frequencies of GC B cells in WT, CD19 KO, and CD19 KO mice that had been reconstituted with WT MZ B cells 2 wk earlier. The mice were cotransferred with MD4 CD45.1^+^ B cells and OTII T cells, followed by immunization with HEL-OVA in Ribi adjuvant. Mice were immunized i.p. and analyzed 8 d later by flow cytometry. (*C*) GC responses in peripheral LNs of WT and CD19 KO mice that had been cotransferred with MD4 and OTII cells and immunized s.c. with HEL-OVA, analyzed at 8 d postimmunization. (*D*) GC B cell responses in CD19 KO and CD19 KO mice reconstituted with WT, CXCR5 KO, or CR1/2 KO B cells as indicated and immunized as above. (*E*) TSS-KI and WT littermate controls were immunized with NP-CGG. Shown are frequencies of NP-specific GC B cells out of the total B cell population. Data from one of three independently performed experiments are presented.

Within minutes after entry to the blood circulation, ICs accumulate in the MZ compartment on the surface of MZ macrophages (10, 12). ICs captured by MZ macrophages are subsequently transferred to local MZ B cells, which carry and deposit them on FDCs inside the follicles (13, 14). We first wanted to test whether this pathway contributes to B cell activation and the establishment of GC B cell responses. To address this question, we used CD19 KO mice, in which endogenous MZ B cells fail to develop (11, 16), as hosts and asked whether these animals can support differentiation of wild-type (WT) antigen-specific B cells into GC B cells. CD19 KO hosts were adoptively transferred with splenocytes from hen egg lysozyme (HEL)-specific B cells (MD4) and ovalbumin (OVA)-specific OT-II T cells and immunized with HEL-OVA–conjugated antigen (17). At 8 d after i.p immunization with HEL-OVA, transferred B cells proliferated and differentiated into GC B cells in the spleens of WT mice, but the response was severely impaired in CD19 KO hosts (Fig. 1 *A* and *B*).

To test the extent to which the absence of MZ B cells contributed to this effect, we reconstituted the MZ B cell compartment by transferring CD19^+^ B cells into CD19 KO hosts. As described previously (10, 11, 18), at 2 to 3 wk after transfer, MZ B cells were detected in the spleens of reconstituted mice at substantial levels (*SI Appendix*, Fig. S1*A*). Reconstitution of the MZ B cell compartment also rescued the GC response (Fig. 1 *A* and *B*). In contrast, when CD19 KO hosts were cotransferred with a large number of WT B cells along with MD4 and OTII cells shortly (1 d) before immunization, the MZ B cell niche was not reconstituted, and the GC response remained impaired (*SI Appendix*, Fig. S1*B*). Notably, peripheral lymph nodes (LNs) of CD19 KO hosts were able to support the proliferation of adoptively transferred MD4 B cells without reconstitution (Fig. 1*C*), further indicating that the defect observed in CD19 KO mice was selective to the spleen, where MZ B cells typically dwell. These findings reveal an important role for MZ B cells in promoting GC B cell differentiation.

### MZ B Cell-Mediated Antigen Shuttling Is Not Required for Their Ability to Support the GC B Cell Response.

Deposition of opsonized antigens on FDCs by MZ B cells depends on the capability of MZ B cells to capture ICs from MZ macrophages and to migrate with them into the follicles. An important pathway that greatly contributes to this process is mediated via the expression of complement receptors CR1/2 on MZ B cells. Removing this pathway has been shown to significantly reduce the capacity of MZ B cells to capture antigenic material and deposit it on FDCs (19–22). Therefore, we asked whether reconstitution of the MZ B cell compartment in CD19 KO hosts with CR1/2 KO B cells will impact their ability to restore the GC response due to suboptimal antigen delivery. CR1/2 KO B cells transferred into CD19 KO hosts developed into MZ B cells (*SI Appendix*, Fig. S1*C*) but were unable to deposit antigen on FDCs (18) (*SI Appendix*, Fig. S1*D*). Surprisingly, however, despite these defects, CR1/2 KO MZ B cells successfully rescued the GC B cell response in CD19 KO hosts, similarly to WT cells (Fig. 1*D*).

While CR1/2 is important for IC capture by MZ B cells, other mechanisms also may contribute to this process, as MZ B cells also express Fc receptors that can bind opsonized antigens (23). Therefore, it was important to test the contribution of MZ B cell-mediated antigen delivery to the development of GC B cell responses using alternative approaches. To transfer their antigenic cargo, MZ B cells must be able to migrate back and forth between the MZ and the follicles, movement that is regulated by interplay with CXCR5 that attracts the cells to the follicular compartment, and S1PR1 that guides them to the MZ (15). Rapid cycles of desensitization and resensitization of S1PR1 drives the cyclical movement of MZ B cells, allowing them to switch direction and alternate the positioning between the MZ and the follicles in a perpetual manner.

To further test whether MZ B cell-mediated antigen shuttling is dispensable for their ability to promote GC B cell responses, we next reconstituted CD19 KO hosts with CXCR5 KO MZ B cells. In line with previous work, in the absence of CXCR5, MZ B cells remained confined to the MZ and were unable to enter the follicles and deposit antigens (13). Again, we found that under these conditions, the GC response was significantly rescued (Fig. 1*D*), suggesting that MZ B cell migration into the follicles is not critical for this process. As a complementary approach to address this question, we also used the S1PR1^TSS^ mouse model. In these animals, a point mutation in the S1PR1 desensitization motif leads to constitutive activity of the receptor, thus retaining the cells in the MZ and preventing them from entering follicles (24). We found that despite defective shuttling of MZ B cells in S1PR1^TSS^ mice, GC B cells developed normally in these mice (Fig. 1*E*). Taken together, these results demonstrate that MZ B cells play an important role in promoting splenic GCs via a mechanism independent of their ability to enter the follicles and deposit opsonized antigens on FDCs.

### SIGN-R1 Macrophages Play a Critical Role in Promoting GC B Cell Responses in the Spleen.

MZ B cells spend approximately one-half of their time in the MZ (18, 25), where MZ macrophages are abundant. Previous studies have shown that homeostatic maintenance of SIGN-R1 macrophages in the spleen depends on the presence of MZ B cells, and that in CD19 KO mice, SIGN-R1^+^ MARCO^+^ MZ macrophages are selectively diminished (*SI Appendix*, Fig. S1*D*) (10, 11). In agreement, we found that the frequencies of SIGN-R1^+^ cells were ∼50% lower in CD19 KO hosts compared with WT mice, a defect that was corrected on reconstitution of the MZ B cell compartment (*SI Appendix*, Fig. S1 *E* and *F*). Therefore, we wondered whether the loss of MZ B cells in CD19 KO hosts may have indirectly compromised GC B cell responses due to impaired SIGN-R1 macrophage function.

To first test the overall role of splenic macrophages in regulating GC B cell responses, we used the previously described mouse model in which the diphtheria toxin receptor (DTR) is expressed under the promoter of CD169. In these mice, administration of diphtheria toxin (DT) leads to rapid depletion of metallophilic as well as MZ macrophage subsets, including both SIGN-R1^+^ MARCO^+^ and SIGN-R1^−^ MARCO^+^ cells (26, 27). CD169-DTR^+/−^ mice were cotransferred as above with MD4 B cells and OT-II T cells and immunized with HEL-OVA–conjugated antigen. As expected, DT-treated mice showed substantial loss of CD169-, MARCO-, and SIGN-R1–expressing cells in the MZ (*SI Appendix*, Fig. S2). Under these conditions, GC B cell frequencies were significantly reduced (*SI Appendix*, Fig. S2). These findings are in agreement with a previous study reporting that short-term clodronate liposomes (CLL) treatment leading to a temporary depletion of all resident macrophage populations in the spleen resulted in similar defects in GC B cell responses (28).

**Fig. 2.**
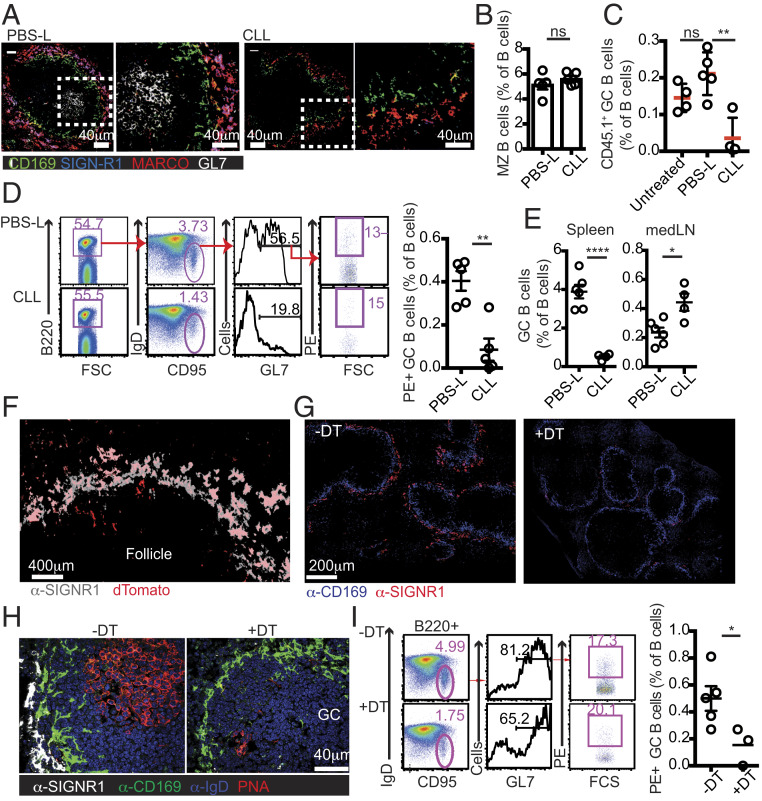
Depletion of SIGN-R1 macrophages impairs GC B cell responses. (*A*–*E*) CLL or PBS-L were infused i.v. to recipient mice at 3 wk before the cotransfer of OTII T cells and CD45.1^+^ MD4 B cells, followed by HEL-OVA immunization. Spleens were analyzed 8 d later. (*A*) IF analysis of immunized PBS-L– and CLL-treated mice. White boxes indicate the high-magnification views shown on the right. (*B*) Frequencies of MZ B cells. (*C*) Frequencies of HEL-specific GC B cells. Data in *A–C* represent one out of at least five independent experiments performed. “Untreated” are immunized mice that did not receive CLL or PBS-L (*D*) Mice were treated with CLL or PBS-L and 3 wk later immunized i.p. with PE in Ribi adjuvant. (*Left*) Gating strategy to determine the frequency of PE^+^ GC B cells. (*Right*) Frequency of PE-specific GC B cells in the B cell compartment at 10 d postimmunization. Data represent one out of three independent experiments. (*E*) GC B cell frequencies in the spleen (*Left*) and mediastinal LNs (*Right*) of mice treated with PBS-L or CLL at 3 wk before intranasal infection with influenza virus. GC B cells were identified at 10 d postinfection as B220^+^IgD^low^FAS^+^GL7^+^ live cells. Data represent one of two independent experiments. (*F*) IF analysis of spleen sections from SIGN-R1-Cre/DTR^+/−^dTomato^+/−^ mice. (*G*) Spleens from SIGN-R1-Cre/DTR^+/−^ mice treated or untreated with DT. (*H* and *I*) GC B cell responses in spleens from DT-treated or untreated SIGN-R1-Cre/DTR^+/−^ mice analyzed at 7 d postimmunization with PE. (*H*) IF analysis with staining as indicated. (*I*) Gating strategy (*Left*) and frequencies (*Right*) of PE-specific GC B cells. Data are from one representative experiment out of three performed. Sections analyzed in *A* and *F*–*H* were from at least three mice per group per experiment.

To define the specific contribution of selected macrophage subsets, we took advantage of the well-established slow recovery rate of SIGN-R1 macrophages after CLL treatment to study a time window in which SIGN-R1^+^MARCO^+^ cells are missing but both SIGN-R1^−^MARCO^+^ and CD169^+^ cells have recovered (7). At 3 wk after CLL administration, mice were transferred with MD4 and OT-II T cells and immunized as above. Control animals were treated with phosphate-buffered saline (PBS)-loaded liposomes (PBS-L), a treatment that had no detectable effect on GC B cell responses (Fig. 2*C*). Immunofluorescence (IF) analysis confirmed the selective absence of SIGN-R1 macrophages, but not of CD169^+^ and MARCO^+^ cells, in CLL-treated mice (Fig. 2*A*). Frequencies of MZ B cells were also similar to those in control-treated animals, confirming their recovery (Fig. 2*B*). Selective depletion of SIGN-R1 macrophages was sufficient to impair the GC response and reduce GC B cell frequencies, reminiscent of the defects observed in DT-treated CD169-DTR animals (Fig. 2*C*).

To gain additional support for the robustness of this effect and to avoid potential biases associated with using transgenic models that are limited to a monoclonal B cell response, we also tested the selective contribution of SIGN-R1 macrophages to the polyclonal B cell response to immunization with other agents, including phycoerythrin (PE) (Fig. 2*D*) and live influenza infection (Fig. 2*E*). In all cases, we found that GC B cell responses in the spleen were dramatically reduced when SIGN-R1 macrophages were missing. In contrast, CLL treatment did not reduce the frequencies of GC B cells detected in mediastinal LNs of influenza-infected mice (Fig. 2*E*), supporting the selective effect of macrophage depletion on the splenic response.

Since the SIGN-R1 receptor can contribute to complement fixation by some antigens (29), we wondered whether the loss of SIGN-R1 itself may have directly contributed to impaired GC B cell responses. To test this, we induced temporal ablation of SIGN-R1 by injecting mice with the 22D1 anti–SIGN-R1 clone, a treatment that down-regulates SIGN-R1 expression in MZ macrophages without ablating the cells (30). Treated mice were immunized with sheep red blood cells (SRBCs) and analyzed 8 d later. Histological analysis of consecutive sections confirmed a selective loss of SIGN-R1 expression in mice treated with 22D1 antibody; however, this treatment did not affect the GC response in the spleen, suggesting that SIGN-R1 itself is not critical for this process (*SI Appendix*, Fig. S3).

### Selective Ablation of SIGN-R1 Macrophages Using a Genetic Mouse Model.

The foregoing results suggested a selective and critical role for SIGN-R1 macrophages in supporting GC B cell differentiation in the spleen. To more directly test this possibility, we generated a knock-in mouse in which a construct expressing Cre recombinase and DTR replaces the SIGN-R1 gene (*SI Appendix*, Fig. S4*A*). To assess the specificity of the model, we crossed the mice to a reporter strain (i.e., Rosa-stop-tdTomato or the Rosa-stop-YFP line). IF analysis showed a substantial labeling of SIGN-R1^+^ cells in the MZ of SIGN-R1^DTR-Cre^^+/−^ tdTomato^+/−^ mice (Fig. 2*F*). Furthermore, treatment with DT led to a considerable loss of SIGN-R1 MZ macrophages (Fig. 2*G*), with no effect on CD169- and MARCO-expressing cells (*SI Appendix*, Fig. S4*B*). As expected (31), in peripheral LNs, we found strong Cre activity primarily in the interfollicular and medullary regions but not in the subcapsular area (*SI Appendix*, Fig. S5*A*), and these signals were greatly reduced on DT treatment (*SI Appendix*, Fig. S5*B*). However, despite the selective targeting of SIGN-R1 macrophages in our SIGN-R1^DTR-Cre^^+/−^ mouse model, in some cases (up to one-third of the mice examined), DT-induced depletion was incomplete. Thus, to test whether DT-treated SIGN-R1^DTR-Cre^^+/−^ mice that had lost SIGN-R1^+^ cells had impaired GC B cell responses, we examined the spleens by IF analysis to confirm ablation, and by flow cytometry to determine frequencies of GC B cells. Consistently, when SIGN-R1 macrophages were efficiently ablated, GC B cell structures were diminished (Fig. 2*H*), and the frequencies of GC B cells dropped significantly (Fig. 2*I*). Thus, these results establish a selective and nonredundant role for SIGN-R1 macrophages in supporting the GC B cell response in the spleen.

### SIGN-R1 Macrophages Are Important for Maturation of the GC Reaction but Not for Initial B Cell Activation and Positioning.

**Fig. 3.**
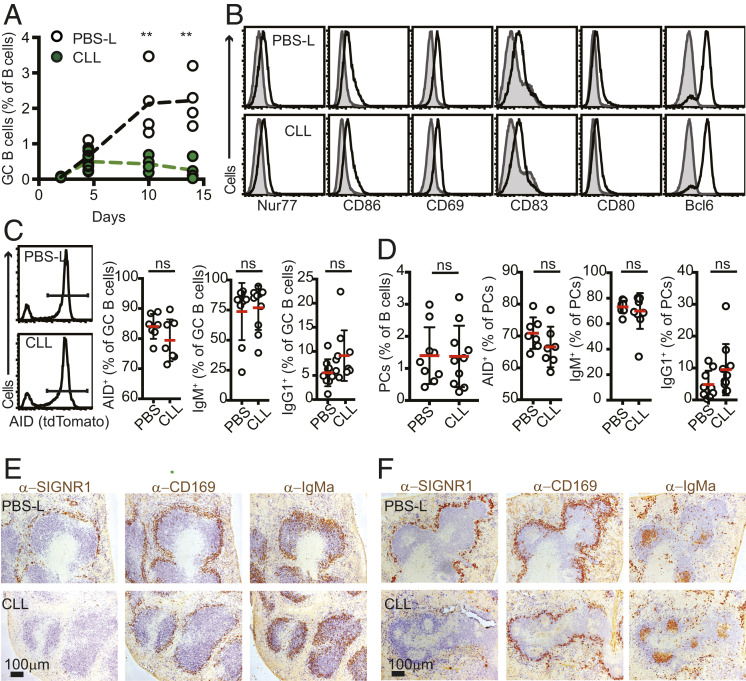
Early development of GC B cells is intact in SIGN-R1 macrophage-depleted mice. (*A*) CLL or PBS-L were injected at 3 wk before i.p. immunization with SRBCs. Spleens were analyzed by flow cytometry at the indicated time points to determine the frequency of GC B cells (B220^+^IgD^low^CD95^+^GL7^+^ live cells) among total B cells. The experiment represents one out of two experiments performed (*n* ≥ 3). (*B*) Flow cytometry analysis of GC B cells from day 5 after SRBC immunization of mice treated with PBS-L (*Top*) or CLL (*Lower*) as above (*n* = 3). The gray histograms show staining in naïve B cells (B220^+^IgD^high^CD95^−^GL7^−^) in immunized animals. Shown are the results of a representative experiment out of three independent experiments performed. (*C* and *D*) AID-tdTomato mice were treated and immunized as above with SRBCs. At 5 d postimmunization, spleens were analyzed. (C) Frequencies of dtTomato^+^, IgM^+^, and IgG1^+^ within the GC B cell pool (pregated on B220^+^IgD^low^FAS^high^GL7^high^). The histograms show examples of tdTomato gating. (D) Frequencies of intracellular staining of IgM and IgG1 within the plasma cell (PC) pool. PCs were defined as B220^low^CD138^+^ cells. Data in *C* and *D* are pooled from two independent experiments. (*E* and *F*) Distribution of HEL-specific activated B cells. Mice were treated with PBS-L or CLL and 3 wk later were cotransferred with HEL-specific CD45.1^+^ MD4 B cells and OTII T cells, followed by i.p. immunization with HEL-OVA. Spleens were collected and snap-frozen at 3 d (*E*) or 5 d (*F*) postimmunization. The distribution of activated B cells was assessed by IHC staining for IgD (to highlight naïve endogenous B cells; blue) and IgM^a^ (to identify transferred MD4 B cells; brown). Selective ablation of SIGN-R1 macrophages, but not of CD169^+^ cells, was confirmed by staining consecutive sections for IgD (blue) and SIGN-R1 or CD169 (brown). The data are from one out of three independent experiments performed. In each experiment, sections from at least three spleens per group were analyzed.

The GC reaction develops over the course of several weeks (32). To first test at which step SIGN-R1 macrophages are necessary, we looked at the kinetics of the GC response in SIGN-R1 macrophage-depleted mice using the MD4 and OTII cotransfer system. At 4 to 5 d postimmunization, frequencies of GC B cells were similar in control and SIGN-R1 macrophage-depleted animals, suggesting intact early priming (Fig. 3*A*). Phenotypically, we could not identify any abnormalities in these cells, as many of the typical markers that are up-regulated in GC B cells at this stage, including Bcl6, CD80, CD83, CD86, Nur77, and CD69, were expressed at normal levels early after activation (Fig. 3*B*). In addition, no defects were detected in the ability of the cells to up-regulate the activation-induced cytidine deaminase (AID) or to isotype switch in newly generated GC B cells and plasma cells (Fig. 3 *C* and *D*). However, while in control animals, GC B cell frequencies continued to increase during the next 9 d, in CLL-treated mice, the response did not progress and appeared to collapse prematurely (Fig. 3*A*). These results point to a requirement for SIGN-R1 macrophages in the later stages of the GC responses and the establishment of a mature GC reaction.

Impaired GC B cell maturation could result from suboptimal activation of B cells that later seed the nascent GC. During the early activation phase, B cells that encounter antigen in the follicles and that receive initial costimulatory signals from CD4 helper T cells migrate to the follicular perimeter (33), where initial proliferation takes place (34). To test whether MZ macrophages play a role in this process, we tracked the synchronized movement of antigen-specific B cells in SIGN-R1 macrophage-depleted mice. At 2 d after immunization, large numbers of antigen-specific B cells (MD4, IgMa^+^) could be identified in the follicular perimeter of control or SIGN-R1 macrophage-depleted animals, indicating intact migration of activated B cells to these sites (Fig. 3*E*). Similar results were obtained when mice were depleted of all MZ macrophage subsets using an acute CLL treatment (*SI Appendix*, Fig. S6).

Migration of B cells to the follicular perimeter depends on activation of the G protein-coupled receptor GPR183 (also known as Epstein–Barr virus-induced gene; EBI2) (35, 36) by its ligand, 7α,25-dihydroxycholesterol (37, 38). To leave these sites and enter the GC structure, the cells down-regulate EBI2. Therefore, signals from macrophages in the MZ compartment might possibly be necessary to induce this step. However, we found no evidence to support this hypothesis; as in the control group, by day 5, activated B cells in CLL-treated mice could leave the follicular perimeter and concentrate in the center of the follicle (Fig. 3*F*).

### Early Activated B Cells Develop Normally in the Absence of SIGN-R1 Macrophages.

**Fig. 4.**
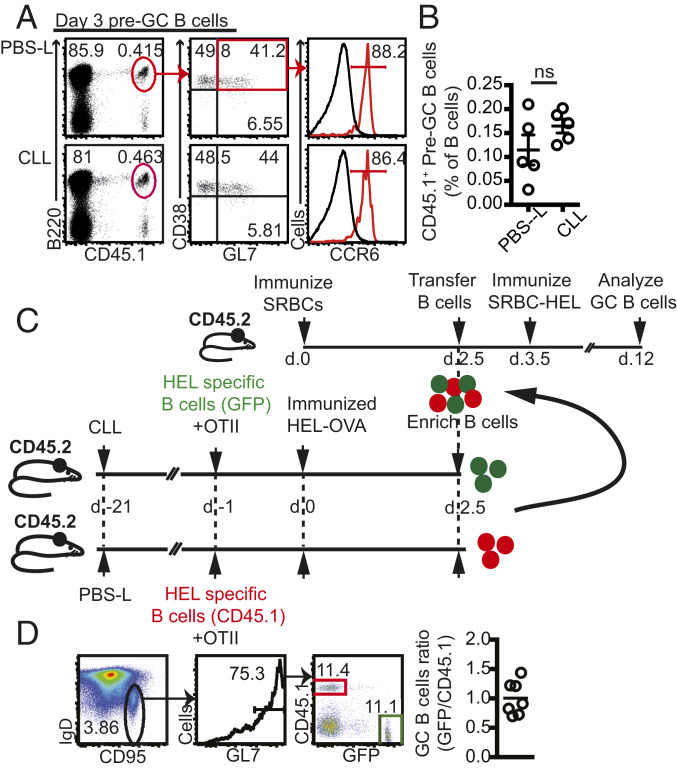
Development of early activated B cells in SIGN-R1 macrophage-depleted mice. (*A* and *B*) CLL or PBS-L were injected i.v., and 3 wk later, MD4 (CD45.1^+^) and OTII cells were cotransferred, followed by i.p. immunization with HEL-OVA. Spleens were analyzed at 3 d postimmunization. (*A*) Representative flow cytometry plots to identify pre-GC B cells. Histograms show CCR6 expression in pre-GC B cells (red) with naïve B cells (gray) as a negative control (gated on B220^+^GL7^−^CD45.2^+^). (*B*) Percentage of CD45.1^+^-specific pre-GC B cells in the B cell compartment. Shown is a representative example out of at least four independent experiments performed. (*C*) Graphic representation of the experimental design. (*D*, *Left*) Flow cytometry plots showing the gating strategy to determine the relative frequencies of transferred CD45.1^+^ and GFP^+^ cells within the GC compartment recovered from recipient mice. Plots were pregated on B220^+^ live cells. (*D*, *Right*) Data pooled from three independent experiments showing the ratio between CD45.1^+^/GFP^+^ cells within the GC compartment after transfer.

The correct positioning of activated B cells during the first 5 d postimmunization in the absence of SIGN-R1 macrophages suggested that these macrophages are not necessary for the initial activation of B cells. In support of this notion, at 3 d after immunization, we found similar frequencies of HEL-specific B220^+^CD38^+^GL7^+^CCR6^+^ B cells in CLL- and PBS-L–treated animals (Fig. 4 *A* and *B*), a population that has been shown to represent a transitional stage between naïve and GC B cells (pre-GCs) (34, 39). To further test whether these pre-GCs are functionally intact, we compared the capacity of pre-GCs that had been primed in SIGN-R1 macrophage-depleted or sufficient hosts to become mature GC B cells. To do this, control and CLL-treated animals received congenically marked HEL-specific B cells (CD45.1^+^ MD4 and GFP^+^ MD4, respectively) together with OTII T cells, followed by immunization with HEL-OVA. At 2.5 d later, spleens were collected, enriched for B cells, and mixed together to obtain a 1:1 ratio between GFP^+^ and CD45.1^+^ B cells. Importantly, the ratio of GFP^+^ and CD45.1^+^ pre-GCs within the transferred B cells was also 1:1 (*SI Appendix*, Fig. S7*A*).

To allow for incorporation of transferred pre-GC B cells into existing GC structures and to provide synchronized T cell help, WT hosts (CD45.2^+^) were immunized with SRBCs 2 d earlier. At 1 d after transfer, recipient mice were boosted with SRBCs conjugated to HEL. Thus, in this system, endogenous SRBC-specific follicular helper T (Tfh) cells provide help to transferred HEL-specific B cells (Fig. 4*C*). Flow cytometry analysis at 8 d after the boost revealed that the ratio between mature GFP^+^ and CD45.1^+^ GC B cells remained at 1:1 (Fig. 4*D*), suggesting that B cell priming in a SIGN-R1 macrophage-depleted environment did not compromise the ability of the cells to mature into GC B cells. These findings further favor a role for SIGN-R1 macrophages in sustaining rather than initiating GC B cell differentiation. In support of this notion, HEL-specific GC B cells that were induced in vitro (iGCs) by incubation of B cells with IL-21 and the 40LB stroma cell line (40) failed to proliferate in SIGN-R1 macrophage-depleted hosts, and their frequencies were significantly lower compared with those recovered from control recipients (*SI Appendix*, Fig. S7 *B* and *C*). Thus, while SIGN-R1 macrophages are dispensable for early activation and migration of B cells, they are essential for the maturation of the response.

### Loss of SIGN-R1 Macrophages Leads to Suboptimal Tfh Cell Activation.

The foregoing results suggest that during the first 5 d of the response, GC B cell development in SIGN-R1 macrophage-depleted mice is intact. Similarly, the frequencies of Tfh cells at this early stage were similar (*SI Appendix*, Fig. S8*A*). We also noted no difference in the expansion of follicular regulatory T cells (CD4^+^ CXCR5^+^ PD-1^+^ FOXP3^+^) (*SI Appendix*, Fig. S8*A*) or in the expression profiles of key known regulatory molecules necessary for the development of Tfh cells (*SI Appendix*, Fig. S8*B*). Furthermore, we found no effect of selective depletion of SIGN-R1 macrophages on the localization of OTII T cells during the early phase of the response, and by day 5 postimmunization, many antigen-specific T cells could be identified at the follicles near nascent GC structures in both control and SIGN-R1 macrophage-depleted mice (*SI Appendix*, Fig. S8*C*).

**Fig. 5.**
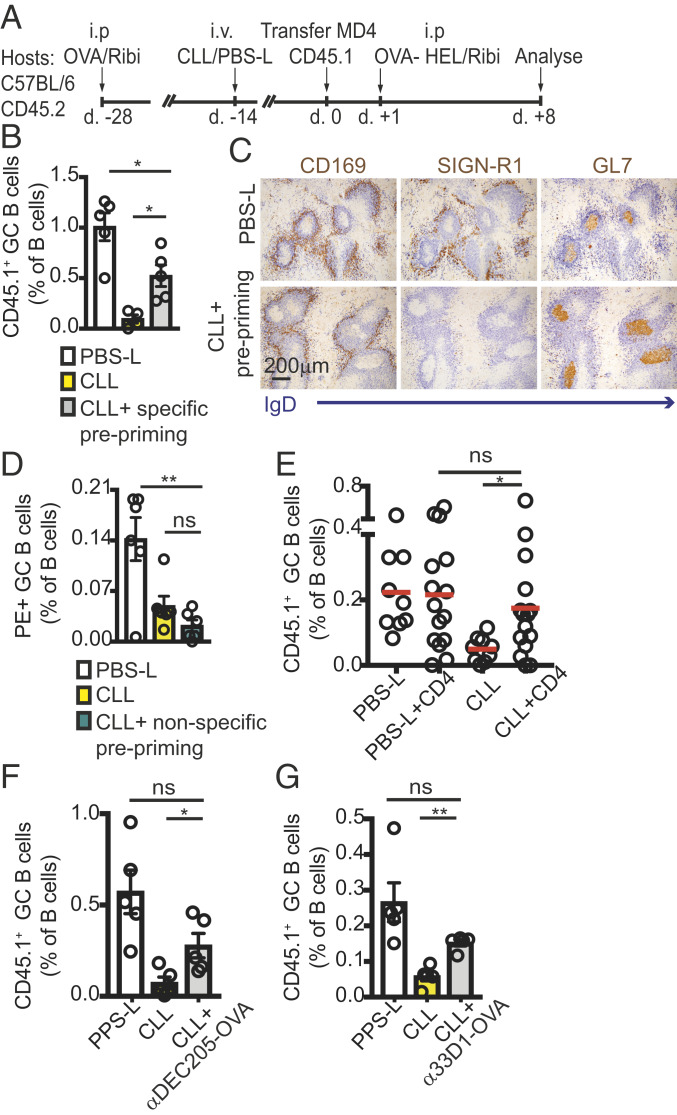
Reconstitution of CD4 T cell priming restores GC B cell responses in SIGN-R1 macrophage-depleted mice. (*A*–*C*) CD45.2^+^ mice were preprimed with OVA at 2 wk before CLL or PBS-L treatment. Two weeks later, mice were transferred with CD45.1^+^ MD4 B cells, followed by immunization with HEL-OVA. Eight days later, spleens were analyzed by IHC and flow cytometry. (*A*) Graphic representation of the experimental protocol. (*B*) IHC analysis at day 8 postimmunization. Sections (from two mice per group) were stained for GL7 (to identify GCs; brown) and IgD (to define follicular B cells; blue). Selective ablation of SIGN-R1, but not of CD169^+^, cells was confirmed by staining consecutive sections for IgD (blue) and SIGN-R1 or CD169 (brown). (*C*) Frequencies of CD45.1^+^ B cells determined by flow cytometry analysis. A representative example is shown out of three independent experiments performed. (*D*) Mice were preprimed with SRBCs 2 wk before CLL or PBS-L treatment, then immunized with PE 2 wk later. Shown are frequencies of PE^+^ GC B cells in splenocytes analyzed by cytometry at 8 d postimmunization, from a representative experiment out of three experiments performed. (*E*) Mice (CD45.2^+^) treated with CLL or PBS-L 3 wk earlier were transferred with CD45.1^+^ MD4 B cells with or without cotransfer of CD4 T cells (CD45.2^+^) that had been purified from mice immunized with OVA 2 wk earlier. Recipients were immunized with HEL-OVA, and spleens were analyzed 8 d later to determine the frequencies of CD45.1^+^ GC B cells. (*F* and *G*) Hosts (CD45.2^+^) were treated with CLLs or PBS-Ls. After 2 to 3 wk, the mice were transferred with CD45.1^+^ MD4 B cells and immunized with HEL-OVA. Then, 3.5 d later, the mice were i.v. infused with or without chimeric anti-DEC205 (*F*) or anti-33D1 (*G*) fused to an OVA-derived peptide. The frequencies of CD45.1^+^ GC B cells were determined 8 d later by flow cytometry. Shown are the results of a representative experiment out of three experiments performed.

Despite our unsuccessful efforts to reveal specific abnormalities in the development of Tfh cells in the absence of SIGN-R1 macrophages, we considered the possibility that any Tfh functional defects might be difficult to reveal using the above assays, as such assays are limited by the choice of phenotypic readouts. As an alternative approach to test the function of Tfh cells, we induced Tfh cells before macrophage ablation. We reasoned that if SIGN-R1 macrophages were necessary for optimal induction of Tfh cells, then generation of Tfh before macrophage ablation should rescue the response. CD45.2^+^ hosts were immunized with OVA, and 2 wk later, mice were treated with CLL or PBS-L to remove macrophages. The hosts were allowed to rest for an additional 2 wk to regain partial recovery of macrophages before receiving MD4 HEL-specific B cells and immunized with HEL-OVA (Fig. 5*A*). Eight days later, we found that the frequencies of GC B cells in CLL-treated mice that were preprimed with OVA were significantly improved compared with animals that were not preprimed (Fig. 5*B*). The selective ablation of SIGN-R1 macrophages and the development of GC B cells in these animals were further confirmed by immunohistochemistry (IHC) analysis (Fig. 5*C*). If prepriming rescued the response by providing CD4 T cell help, we expected induction of irrelevant Tfh cells to have no effect. Therefore, we next preprimed mice with SRBCs. We used SRBCs to drive a strong response in an attempt to reveal possible effects of nonspecific priming. At 2 wk after macrophage ablation, mice were immunized with PE, and 8 d later, the frequencies of PE-specific GC B cells were determined by flow cytometry. Prepriming mice with an irrelevant antigen before macrophage depletion did not rescue the response and did not help drive PE-specific B cells in CLL-treated mice (Fig. 5*D*).

Taken together, the foregoing findings support the possibility that SIGN-R1 macrophages may be necessary to achieve optimal induction of Tfh cells, and that in their absence, GC failure is at least partially due to insufficient T cell help. In agreement with this possibility, we found that CD4 T cells that were primed in control animals and transferred into SIGN-R1 macrophage-depleted hosts significantly improved the recipient GC response (Fig. 5*E*). Furthermore, enhancing antigen presentation to Tfh by injecting anti-DEC205 (41, 42) or anti-33D1 antibody (43) conjugated to an OVA peptide at 3.5 d after immunization led to a partial, but significant, increase in the frequencies of GC B cells in SIGN-R1 macrophage-depleted mice (Fig. 5*F*). Taken together, these results demonstrate that defects in maintaining GC B cell responses in SIGN-R1–depleted mice can be partially compensated for by boosting Tfh activation.

### SIGN-R1 Macrophages Regulate Positioning of DCIR2^+^ DCs in the Bridging Channels.

**Fig. 6.**
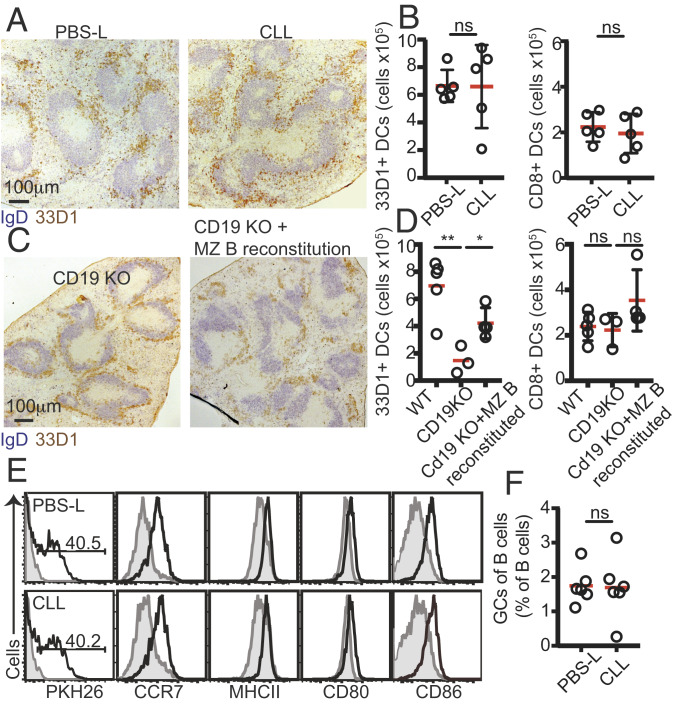
Positioning of DCIR2^+^ DCs in the interfollicular regions depends on SIGN-R1 macrophages and is necessary for GC responses. (*A*) Histological analysis of frozen spleen sections stained with IgD (blue) and 33D1 (brown) of mice treated with PBS-L or CLL 3 wk earlier. (*B*) DC subsets in mice treated with PBS-L and CLL as above. (*C*) Histological analysis of frozen spleen sections stained as in *A* from CD19 KO mice that had or had not been transferred with WT B cells to reconstitute the MZ B cell compartment. (*D*) DC subsets in CD19 KO and CD19 KO reconstituted mice. (*E*) Mice that had been treated with PBS-L or CLL 2 to 3 wk earlier as above were immunized with PKH26-labeled SRBCs, and 3 h later, spleens were harvested and analyzed by flow cytometry. Shown is a representative example out of three experiments performed. (*F*) Mice that had been treated with PBS-L or CLL 3 wk earlier were immunized with VLPs and analyzed by flow cytometry 11 d later. GC B cells were identified as B220^+^IgD^low^CD95^high^GL7^+^ live cells. Data are shown from at least one out of three independent experiments. In *A* and *C*, sections from at least three spleens were analyzed in each experiment.

SIGN-R1 macrophages are located at the outer rim of the MZ, outside the white pulp. As such, these cells are unlikely to come in direct contact with CD4 T cells. Therefore, we considered the possibility that SIGN-R1 macrophages may instead be regulating Tfh cell activation indirectly, by modulating other factors necessary for this process. DCs play key roles in presenting antigens to T cells and promoting the development of Tfh cells. In the spleen, the main DC subset that activates CD4^+^ T cells is characterized by expression of DCIR2 and CD4 (44). Under homeostatic conditions, the chemoattractant receptor EBI2 regulates the localization of these cells at the bridging channels (45, 46) in close proximity to the MZ compartment, where SIGN-R1 macrophages are abundant. Positioning of DCIR2^+^ DCs at these sites is necessary for the survival of the cells and for their ability to prime CD4^+^ T cells and promote GC B cell responses (45, 46), a defect that can be largely compensated by immunization with anti–33D1-OVA (46). To test whether SIGN-R1 macrophages are necessary for DCIR2^+^ DC localization at the bridging channels, we looked at their distribution in the spleens of macrophage-sufficient and -depleted mice. Histology analysis revealed that when SIGN-R1 macrophages were depleted, DCIR2^+^ DCs were no longer clustered at the bridging channels (Fig. 6*A*); instead, the cells were displaced to the MZ compartment, forming a ring that occupied the outer rim of the MZ. However, despite being important for their survival, displacing DCIR2^+^ DCs from the bridging channels did not lead to consistent changes in their numbers, as determined at 2 to 3 wk after CLL treatment (Fig. 6*B*). This could be due to the relatively short time frame of the assay. To test this possibility, we looked at the distribution and frequency of DCIR2^+^ DCs in CD19 KO mice, where the absence of MZ B cell development leads to long-term defects in the maintenance of SIGN-R1 macrophages (10, 11). In agreement with previous studies, CD19 KO mice showed abnormal distribution of DCs (10, 11), with DCIR2^+^ DCs redistributed to the MZ (Fig. 6*C*). Under these conditions, the number of DCIR2^+^ DCs, but not CD8^+^ DCs, in the spleens of CD19 KO mice was significantly reduced (Fig. 6*D*). On reconstitution of the MZ B cell niche and recovery of the overall architecture of the compartment (11, 32), the positioning of DCIR2^+^ DCs was corrected, and the number of DCIR2^+^ DCs was significantly increased (Fig. 6 *C* and *D*). Displaced DCIR2^+^ DCs were able to quickly capture PKH26-labeled SRBCs and to increase expression levels of activation molecules, including CCR7, MHC class II, CD80, and CD86, in a manner similar to that seen in control-treated animals (Fig. 6*E*). Taken together, these observations demonstrate that SIGN-R1 macrophages are important for DCIR2^+^ DC positioning at the bridging channels but not for their activation, and show that MZ B cells are necessary to maintain this function.

Recent work has shown that during immunization with nanoparticle antigens, such as virion-like particles (VLPs), the priming of Tfh cells is independent of DCs and is instead dominated by antigen-specific B cells (33). Thus, if SIGN-R1 macrophage-depleted mice fail to mount a mature GC response due to impaired priming of Tfh cells owing to a loss of DCIR2^+^ DC positional regulation, applying a DC-independent type of immunization should bypass this defect and rescue the GC response. Indeed, SIGN-R1 macrophage-depleted animals that were immunized with VLPs mounted a similar GC response as seen in control animals (Fig. 6*F*).

## Discussion

MZ macrophages are composed of a heterogeneous mixture of cells containing at least two subsets, one that can be identified by the expression of the scavenger receptor MARCO and the lectin receptor SIGN-R1 and another that lacks SIGN-R1 expression. Here we used a combination of approaches to investigate the specific contribution of SIGN-R1 macrophages to the regulation of humoral immunity. We found that GC B cell responses were severely impaired when SIGN-R1 macrophages were selectively absent following CLL treatment. Similarly, in CD19 KO mice, in which lack of MZ B cell development leads to selective loss of SIGN-R1 macrophages, GC B cell responses failed to develop. Importantly, these findings were confirmed using our newly generated SIGN-R1^Cre/DTR^ KI mouse model in which SIGN-R1 cells are directly and selectively depleted by DT treatment.

Notably, while administration of ablating substances, such as CLL and DT, is associated with systemic depletion of macrophages, loss of SIGN-R1–expressing cells in CD19 KO hosts is selective to the spleen, where MZ B cells reside, providing important support to the contribution of local SIGN-R1 MZ macrophages to the response.

Previous studies have shown that MZ B cells and SIGN-R1 macrophages are engaged in a reciprocal cross-talk, and that this axis is important for the regulation of both cell types. When MZ B cells were absent, a significant and selective loss of SIGN-R1 macrophages was observed, which led to reduced uptake of the polysaccharide Ficoll from the blood. Subsequently, deposition of Ficoll in the follicles was impaired (10–12). Thus, MZ B cells can regulate the composition of macrophages in the MZ compartment, which in turn affects their own capacity to deliver antigenic material to the follicle. However, the extent to which antigen transport via this pathway drives activation of follicular B cells and promotes subsequent formation of GC B cell responses has not been tested previously. In this study, we addressed this question. We found that despite the efficient manner by which MZ B cells carry opsonized antigens into the follicles, interfering with this process by preventing MZ B cell migration or by reducing their ability to capture ICs did not impair the development of GC B cells. These observations may reflect a significant level of redundancy in the system and the likely existence of alternative routes by which antigenic material can be delivered into the splenic follicles.

Notably, unlike in LNs, where circulating cells and lymph-born antigens enter the tissue via different routes and first come in contact in the follicles (47), in the spleen, both lymphocytes and systemic antigens arrive together with the blood flow and may come in contact during the entry process. Therefore, it is possible that multiple cells types that pass via this compartment are able to detect and respond to antigenic material, allowing for a greater degree of compensation between partially overlapping pathways.

We currently do not know whether splenic SIGN-R1 macrophages also contribute to prolonging the mature GC reaction. Experiments attempting to address this question by depleting macrophages at later stages of the response were compromised by an overwhelming inflammatory response that was induced shortly after macrophage ablation. Specific targeting of selected relevant genes without involving cellular depletion may help address this point directly.

A role for MZ and metallophilic macrophages in regulating the migration of activated B cells to the perimeter of the follicles has been proposed (28). However, in the present study, we did not observe any such migratory defects, and in macrophage-depleted mice, activated B cells were able to localize to the follicular perimeter at 2 to 3 d after challenge, similar to control animals. It is possible that the discrepancy between the studies is related to the manner of CLL administration; while in the previous study, mice were immunized at 2 to 4 d after CLL injection, we allowed longer recovery times after CLL administration. Our observation that activated B cells are able to migrate normally to these sites favors the notion that metallophilic and MZ macrophages are not a dominant source of 7α,25-dihydroxycholesterol (7α,25-HC), the key EBI2 ligand involved in the attraction of activated B cells to these sites (38, 48). This is in agreement with previous studies demonstrating that splenic 7α,25-HC is produced mainly by stromal cells (49). Thus, it is possible that SIGN-R1 macrophages may instead be necessary for preventing DCIR2^+^ DCs from entering the MZ compartment, or that their removal may induce local cues that trigger migration to these sites. Additional studies are needed to address these possibilities and to define the precise mechanisms by which SIGN-R1 macrophages regulate DCIR2^+^ DC positioning in the spleen.

Our study shows that in the absence of SIGN-R1 macrophages, DCIR2^+^ DCs lose their clustered positioning in the bridging channels and are instead displaced to the MZ. A similar phenotype was previously reported in mice treated with FTY720 (50), in which MZ B cells are displaced into the follicles (25), a condition that correlates with a transient loss of SIGN-R1 macrophages (10, 11). Since localization of DCIR2^+^ DCs at the bridging channels is essential for their survival and ability to drive optimal Tfh responses (48), displacement of DCIR2^+^ DCs from these sites may contribute to the impaired GC B cell responses observed in SIGN-R1–depleted mice. In support of this possibility, we found that in CD19 KO animals, restoring the SIGN-R1 macrophage compartment by reconstituting the MZ B cell niche restored DCIR2^+^ DC positioning at the bridging channels and rescued the GC B cell response. We further found that induction of a specific Tfh cell response before macrophage ablation or boosting Tfh cell activation during later stages of the response significantly rescued the development of GC B cells in SIGN-R1 macrophage-depleted mice. Finally, we found that bypassing the need for DC-mediated Tfh cell priming by immunizing mice with VLPs, a form of immunization that has been shown to rely on B cell-mediated Tfh cell priming (51), was sufficient to induce a GC B cell response of normal magnitude in the absence of SIGN-R1 macrophages. This study provides evidence that altered priming of Tfh cells can lead to defects in their ability to support long-lasting GC responses. While further work is needed to define the defects in the Tfh cells, our study highlights how the targeting of vaccine antigens to appropriate antigen-presenting cells is likely to be critical for mounting robust antibody responses.

## Material and Methods

### Mice.

C57BL/6 (B6, CD45.2^+^) and B6 Ly5.2 (CD45.1^+^) mice were purchased from Charles River Laboratories. MD4 (52) (MGI: 2384162) and OTII (53) (MGI: 2174541), have been described previously. CD19 KO mice were in a B6 background and were generated by intercrossing CD19 Cre^+/+^ (54) mice to obtain CD19 Cre/Cre mice. Mice expressing GFP under the human ubiquitin promoter (Ub-GFP, 004353; Tg(UBC-GFP)30Scha/J), CXCR5 KO (55) (MGI: 2158677), AID-Cre (007770; B6.129P2Aicdatm1(cre)Mnz/J), Rosa26-stop-tdTomato (007914; B6.Cg-Gt(ROSA)26Sortm14(CAG-tdTomato)Hze/J, and Rosa26-stop-YFP (006148; B6.129 × 1-Gt(ROSA)26Sortm1(EYFP)Cos/J) were purchased from The Jackson Laboratory. Mice expressing the DTR under the CD169 promotor (CD169-DTR mice) were described previously (26, 27) and purchased from the RIKEN BioResource Center. CR1/2 KO mice (56) and S1PR1^TSS^ knockin mice (24) were described previously. SIGN-R1-Cre/DTR mice were generated by Biocytogen using B6 ES cells. In these mice, a cDNA construct encoding an Frt-flanked neomycin resistance cassette followed by a stop cassette was introduced before a start codon, followed by cDNA encoding Cre recombinase, and the human DTR (HBEGF) was generated. The Cre recombinase and DTR coding sequences were separated by self-cleaving 2A viral sequences to allow stoichiometric translation of both individual proteins. This construct was inserted at the ATG start codon of cd209b (SIGN-R1), effectively replacing the expression of SIGN-R1. To remove the neomycin resistance gene and stop cassette, homozygous mice were crossed with B6.Cg-Tg(Pgk1-FLPo)10Sykr/J (The Jackson Laboratory), which expresses the mouse codon-optimized FLP recombinase under the direction of the mouse Pgk1 (phosphoglycerate kinase 1) promoter.

Mice were bred and maintained under specific pathogen-free conditions in accredited animal facilities at the Kennedy Institute of Rheumatology, University of Oxford or at the University of California San Francisco. All experiments were conducted in accordance with the UK Scientific Procedures Act (1986) under a project license authorized by the UK Home Office and in accordance with protocols approved by the University of California San Francisco’s Institutional Animal Care and Use Committee.

### Cell Isolation, Adoptive Transfer, and Immunization.

GC B cell responses were tracked using MD4 B cells and OTII T cells as described previously (57). In brief, the frequency of HEL-specific Tg B cells (determined as B220^+^IgMa^+^HEL^+^) and the frequency of OVA-specific T cells (determined as CD4^+^Vα2^+^Vβ5^+^) were first assessed by flow cytometry to determine concentration, followed by cotransfer of ∼2 × 10^5^ HEL-specific B cells (referred to as MD4) and 2 × 10^5^ OVA-specific CD4 T cells (OTII) into recipient mice. The next day, mice were injected intraperitoneally (i.p) with 50 μg of conjugated HEL-OVA in Ribi.

In some experiments, CD4 T cells from immunized donor mice were transferred into recipients. In these experiments, donor C57BL/6J CD45.1^+^ mice were immunized with OVA in Ribi. At 2 wk later, spleens were collected, and CD4 T cells were enriched by incubating the cells for 30 min with antibodies against B220 (RA3-6B2), CD11b (M1/70), CD11c (N418), MHCII (M5/114.15.2), Ter119, and Ly6G (1A8). Cells were then washed with RPMI and incubated with anti-rat IgG magnetic beads (Qiagen; 310107) for 30 min on a rotator at 4°, followed by negative selection using a magnet (Life Technologies; 12301D).

For visualization of in situ B cell position at day 2 postimmunization, B6 (IgM^b^) mice were adoptively transferred with splenocytes containing 10 to 20 × 10^6^ MD4 (IgM^a^) B cells and 5 to 10 × 10^6^ OTII T cells. At 1 d after cell transfer, recipients were immunized with an i.p. injection of 50 μg of conjugated HEL-OVA in Ribi. Between 2 and 5 d later, spleens were harvested and analyzed by IHC for poisoning of IgM^a^ cells.

To examine the distribution of CD4 T cells, CD4 T cells were enriched from the spleens of CD45.2^+^ OTII mice by negative selection using B220, IgD, Ly6G, and CD11b antibodies, followed by anti-rat beads and magnetic purification as above. Each recipient B6 CD45.1^+^ mouse received ∼6 × 10^6^ enriched OTII CD4 T cells and splenocytes containing 6 × 10^6^ CD45.1^+^ MD4 B cells. One day later, CD45.1^+^ recipients were immunized with HEL-OVA in Ribi, and the spleens were collected and snap-frozen on day 5. The localization of OTII transferred T cells was determined by IHC analysis by staining for CD45.2.

### Reconstitution of the MZ B Cell Compartment.

To reconstitute the MZ B cell compartment for immunization assays, purified B cells from B6, CD1/2 KO, or CXCR5 KO mice (10 to 15 × 10^6^) were transferred to CD19 KO recipient mice for 2 to 3 wk before MD4 B cell and OTII T cell transfer and HEL-OVA immunization, as described above.

### Immunization and Conjugation of SRBC-HEL and SRBC-PKH26.

Unless stated otherwise, immunizations were administered i.p. in a volume of 200 μL. For immunization with HEL-OVA and OVA, Ribi adjuvant (Sigma-Aldrich; S6322) was reconstituted in 1.1 mL of PBS and mixed 1:1 with 0.5 mg/mL protein in PBS. For immunization with PE (Thermo Fisher Scientific; P801) and NP-CGG (Biosearch Technologies; N-5055C), 50 μg of the antigen was mixed at a 1:1 ratio with alum adjuvant and rotated for 10 min before injection. For immunization with SRBCs, 1 mL of sterile SRBCs (Thermo Fisher Scientific; 12977755) were washed once with 50 mL of PBS and then reconstituted in 2 mL of PBS. For immunization with SRBC-HEL, 1 mL of sterile SRBCs were washed three times in PBS and once with 10 mL of conjugation buffer (0.35 M mannitol [Sigma-Aldrich; M4125] and 0.01 M NaCl) with the brake off. Cells were reconstituted in 0.9 mL of conjugation buffer and the mixed with 20 μg/mL HEL. The solution was mixed for 10 min on ice, followed by the addition of 10 mg of EDC [1-ethyl-3-(3-dimethylaminopropyl)carbodiimide hydrochloride] (Thermo Fisher Scientific; 22980) to a final volume of 1 mL, followed by 30 min of rocking gently on ice. The cells were washed four times in PBS and resuspended in final volume of 2 mL of PBS (52). For immunization with VLPs, 25 μg of VLPs were injected i.p. Qbeta VLPs were generated using a prokaryotic expression vector based on pGEM encoding the Qbeta coat protein (pQb10), transformed into *Escherichia coli* JM109 cells (Sigma-Aldrich), as described previously (58).

For immunization with SRBCs conjugated to PKH26, fresh SRBCs were conjugated to PKH26 (Sigma-Aldrich) according to the manufacturer’s instructions with 10 μL of PKH26 dye (1 mM) per 1 mL of blood cells resuspended in 1 mL of conjugation buffer. Approximately 200 × 10^6^ PKH26- conjugated SRBCs were injected i.v. at 3 h before analysis by flow cytometry, as described previously (59).

### Macrophage Depletion.

CLLs or PBS-loaded control liposomes were purchased from Liposoma BV or Encapsula NanoSciences and were administered i.v. according to the manufacturer’s instructions. To deplete macrophages in CD169-DTR or in SIGN-R1-Cre/DTR mice, DT (Merck KGaA**)** was infused i.v. at 30 ng/g of body weight at 6, 4, and 1 d before immunization. The administration of DT was spread out over the course of 7 d before immunization to limit the effect of acute cell death of a large number of cells. We found that this DT administration schedule did not lead to any detectable inflammatory effects at the time of immunization. An additional DT injection was given at 3 d after immunization to ensure maintenance of SIGN-R1 macrophage depletion throughout the response.

### In Vivo Antibody Treatments and Production of Anti-DEC205-OVA and Anti-33D1-OVA.

To induce temporal depletion of SIGN-R1, B6 mice received one i.v. injection of 100 μg of anti–SIGN-R1 antibody (22D1; Bio X Cell) or control hamster antibody (PIP; Bio X Cell). One day later, mice were cotransferred with MD4 B cells or OTII T cells, followed by immunization with HEL-OVA.

The generation of anti-DEC205-OVA conjugated antibody has been described previously (60). In brief, HEK293T cells were grown in a 10-cm dish in DMEM supplemented with 10% FBS and 10 mM Hepes and then transfected with plasmids encoding the heavy and light chains of DEC205-Ova antibody using Lipofectamine 2000 (Thermo Fisher Scientific; 11668019). On days 1 and 4 after transfection, the medium was exchanged with fresh medium. On days 4 and 6, the supernatant was collected, spun to remove cell debris, and adjusted to pH 7.0. The antibody was purified using an HiTrap GHP column (Sigma-Aldrich; 29-0485-81) according to the manufacturer’s instructions. The product size was confirmed by SDS/PAGE. Anti–33D1-OVA was produced similarly in 293T cells transduced with anti–33D1-OVA plasmid (43) and purified through protein G affinity chromatography. Mice were infused i.v. with 10 μg of purified anti–DEC205-OVA or 2 μg of purified anti–33D1-OVA.

### Generation and Adoptive Transfer of In Vitro-Induced GC B Cells and In Vivo-Induced Pre-GCs.

To induce GC B cells in vitro, CD45.1^+^ MD4 B cells were grown on irradiated (60 Gy) 40LB cells supplemented with rIL-4 (1 ng/mL; eBioscience; 34-8041-85), as described previously (40). The 40LB cell line was a kind gift from Daisuke Kitamura. Six days later, B cells were harvested and analyzed by flow cytometry to confirm GC B cell phenotype (live B220^+^IgD^low^FAS^+^GL7^+^). Induced GC B cells (2 to 3 × 10^6^) were subsequently transferred into CD45.2^+^ recipient hosts.

To induce pre-GCs in vivo, CD45.2^+^ B6 mice were treated with CLL or PBS and 3 wk later were cotransferred with 5 to 6 × 10^6^ OTII T cells together with 5 to 6 × 10^6^ GFP^+^ or 5 to 6 × 10^6^ CD45.1^+^ MD4 B cells. The next day, the mice were immunized with HEL-OVA. On day 2 postimmunization, spleens were collected and enriched for B cells using the CD43 MicroBeads Kit (Miltenyi Biotec; 130-049-801) according to the manufacturer’s protocol. The frequencies of GFP^+^ and CD45.1^+^ pre-GC B cells of each immunized mouse were analyzed by flow cytometry (defined as live B220^+^CD38^+^GL7^+^CCR6^+^). Equal numbers (1.5 × 10^5^) of GFP^+^ pre-GC B cells and CD45.1^+^ pre-GC B cells (derived from CLL- and PBS-treated mice, respectively) were mixed and analyzed once more by flow cytometry to ensure a 1:1 ratio before being transferred into CD45.1^+^ B6 recipients.

### Flow Cytometry.

Spleens were collected in RPMI-1640 (2% FBS and 0.01 M Hepes) and mashed through a 40-μm mesh. Cells were incubated with antibodies for 20 min on ice in FACS buffer (PBS, 1 mM EDTA, 0.1% azide, and 2% FBS). Samples were acquired with an LSR Fortessa or LSR-II flow cytometer (BD Biosciences). Data were analyzed using FlowJo software. The following antibodies were used: B220 (RA3-6B2), IgD (11-26c-2a), CD23 (B3B4), CD1d (1B1), CD21/35 (7E9), CD45.1 (A20), GL7, FAS (Jo2), Nur77 (12.4), CD86 (GL-1), CD69 (H1.2F3), CD83 (Michel-19), CD80 (16-10A1), Bcl6 (BCL-DWN), CD38 (90), CCR6 (140706), CD138 (281-2), and CCR7 (4B12). Fixable Viability Dye eFluor 780 (eBioscience) was used to stain dead cells, and PE (Thermo Fisher Scientific) was used to label PE-specific GC B cells. A Foxp3 transcription factor staining buffer set (eBioscience) was used for transcription factor staining.

### Histology Staining and Analysis.

Spleens and LNs were embedded in optimum cutting temperature (OCT) compound (Agar Scientific; AGR1180) and snap-frozen in dry ice with 100% methanol, after which 7-μm sections were cryostat-cut and collected on SuperFrost Plus Adhesion slides (Thermo Fisher Scientific). Sections were fixed in cold 100% acetone for 10 min and then allowed to dry for at least 1 h. Before staining, slides were rehydrated for 10 min in PBS (for IF analysis) or Tris-buffered saline (for IHC analysis) and blocked with normal mouse serum (2%) for 20 min in a humidified chamber. Primary antibody staining was performed for 2 to 3 h, followed by secondary staining for 1 h. For IF analysis, slides were mounted with Fluoromount-G (Cambridge Bioscience). IF images were captured with an Olympus FV1200 confocal microscopy system. For IHC analysis, slides were mounted with Aqua-Mount (Thermo Fisher Scientific) and imaged with a Zeiss Axio Scope.A1. The following antibodies were used: Armenian hamster SIGN-R1 (22D1; eBioscience), CD169-FITC (3D6.112; Bio-Rad), MARCO (ED31; Bio-Rad), GL7-FITC (BioLegend), GL7-bio (eBioscience), PNA-bio (Sigma-Aldrich), IgD-FITC (11-26c.2a; BioLegend), IgD-bio (11-26c; eBioscience), anti–hamster-APC (BioLegend), anti–hamster-HRP (The Jackson Laboratory), anti–hamster-AP (The Jackson Laboratory), anti–rat-Cy3 (Stratech), SA-Cy3 (Thermo Fisher Scientific), anti–FITC-A488 (The Jackson laboratory), anti–FITC-HRP (Roche Applied Science), and SA-AP (The Jackson Laboratory). For imaging of dTomato in SIGN-R1-Cre/DTR Tomato^f/f^ mice, spleens were cut into quarters and incubated in 4% PFA in PBS for 2 h at 4 °C, washed three times with cold PBS for 5 min, dehydrated in 30% sucrose overnight, and embedded in OCT compound.

To analyze the relative frequency of SIGN-R1–expressing cells in the spleens of WT, CD19 KO, and CD19 KO mice reconstituted with MZ B cells, spleens were harvested and stained as above with anti–SIGN-R1 antibody. Large tiles of sections were imaged using confocal microscopy. Surfaces surrounding the SIGN-R1–positive cells in each section were created automatically using Imaris software. The total area covered by surfaces and the total area of the section (μm^2^) were calculated using Imaris, and the ratio between them was plotted.

### PE-IC Deposition on FDCs.

PE-ICs were induced by passive immunization of mice with 2 mg of polyclonal rabbit anti-PE (200-4199; Rockland), followed 2 h later by injection of 20 μg of PE (P-801; Invitrogen) as described previously (61). Some 16 h later, spleens were collected and then frozen in OCT compound (Agar Scientific) for IF analysis (18).

### Statistical Analysis.

Unless stated otherwise, *P* values were calculated with unpaired two-tailed Student’s *t* test for two-group comparisons. Data are displayed as mean ± SD. A *P* value <0.05 was considered significant; and the following values were delineated: *P < 0.05, **P < 0.01, ***P < 0.001, and ****P < 0.0001. Statistical analysis was performed with GraphPad Prism 7.

### Data Availability.

All data, associated protocols, methods, and sources of materials are available in the main text or *SI Appendix*.

## Supplementary Material

Supplementary File
