# Supplementary material for: Marginal zone SIGN-R1+ macrophages are essential for the maturation of germinal center B cells in the spleen
Source: Proc Natl Acad Sci U S A. 2020 May 18;117(22):12295–305. doi: 10.1073/pnas.1921673117 (PMC7275705; doi:10.1073/pnas.1921673117)
Supplement: Supplementary File [file pnas.1921673117.sapp.pdf]

PNAS
www.pnas.org

Supplementary Information for

Marginal zone SIGN-R1$^+$ macrophages are essential for the maturation of germinal centre B cells in the spleen

Gabriela Pirgova[a], Anne Chauveau[a], Andrew J MacLean[a], Jason G Cyster[b], Tal I Arnon[a]

Corresponding author: Tal Arnon

Email: tal.arnon@kennedy.ox.ac.uk.

This PDF file includes:

Figures S1 to S8

www.pnas.org/cgi/doi/10.1073/pnas.1921673117

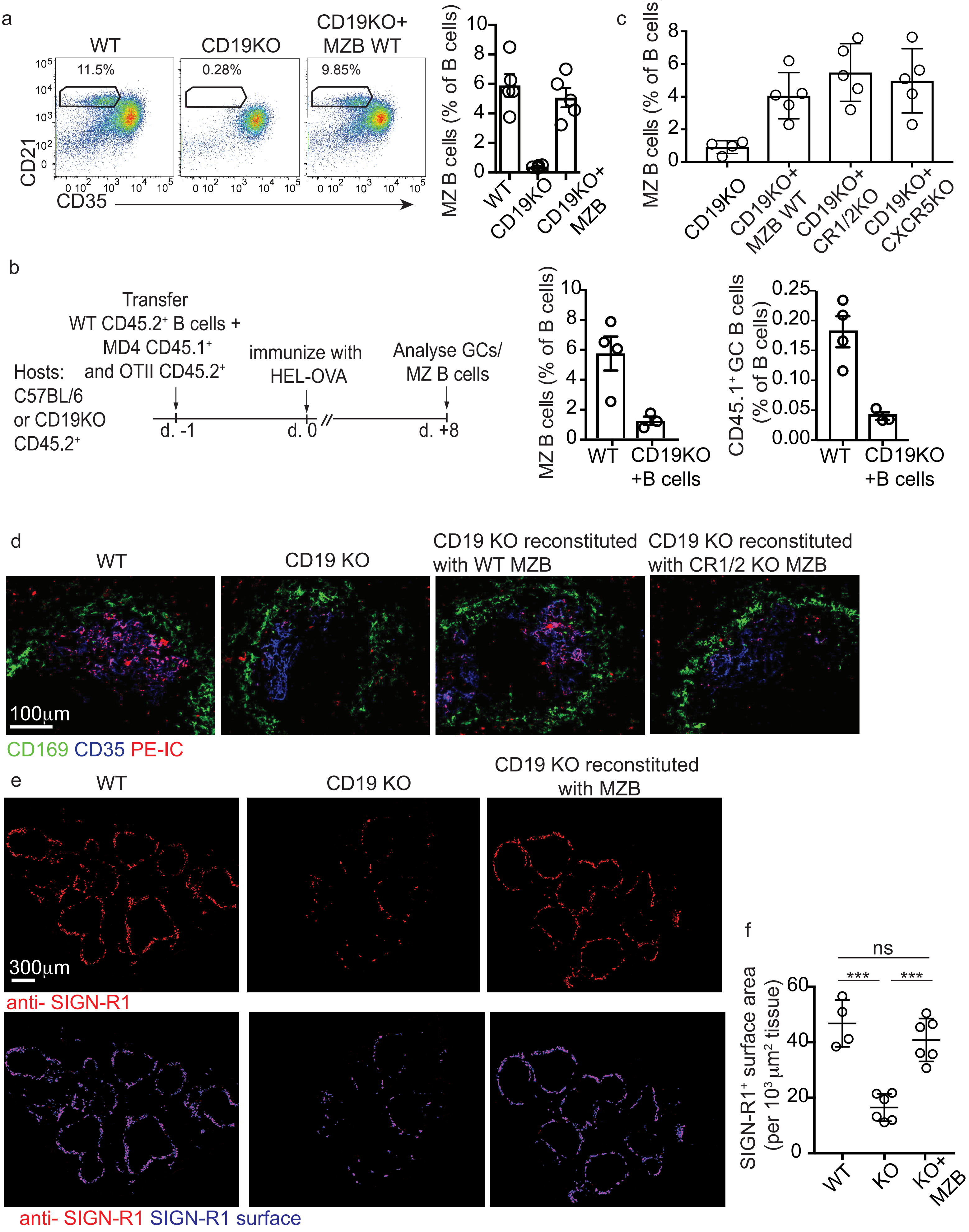
a
WT
CD19KO
CD19KO+
MZB WT
11.5%
0.28%
9.85%
CD21
CD35
MZ B cells (% of B cells)
WT
CD19KO
CD19KO+
MZB
c
MZ B cells (% of B cells)
CD19KO
CD19KO+
MZB WT
CD19KO+
CR1/2KO
CD19KO+
CXCR5KO
b
Transfer
WT CD45.2⁺ B cells +
MD4 CD45.1⁺
and OTII CD45.2⁺
Hosts:
C57BL/6
or CD19KO
CD45.2⁺
immunize with
HEL-OVA
Analyse GCs/
MZ B cells
d. -1
d. 0
d. +8
MZ B cells (% of B cells)
WT
CD19KO
+B cells
CD45.1⁺ GC B cells
(% of B cells)
WT
CD19KO
+B cells
d
WT
CD19 KO
CD19 KO reconstituted
with WT MZB
CD19 KO reconstituted
with CR1/2 KO MZB
100μm
CD169 CD35 PE-IC
e
WT
CD19 KO
CD19 KO reconstituted
with MZB
300μm
anti- SIGN-R1
anti- SIGN-R1 SIGN-R1 surface
f
ns
***
***
SIGN-R1⁺ surface area
(per 10³ μm² tissue)
WT
KO
KO+
MZB

Figure S1

Figure S1. Reconstitution of MZ B cells in CD19 KO mice

(a) Left, flow cytometry plots showing MZ B cell gate in WT, CD19 KO and CD19 KO mice that have been reconstituted with WT B cells. Plots were pre-gated on live, B220+ cells. Right, frequencies of MZ B cells. (b) On the left, graphic representation of experimental protocol. On the right, frequencies of MZ B cells and GC B cells in WT, CD19 KO or CD19 KO mice that had been transferred with 10^6 WT B cells together with MD4 B cells and OTII T cells one day before immunization with HEL-OVA. Frequencies of GC and MZ B cells were analysed 8 days later by flow cytometry. (c) Frequencies of MZ B cells detected in CD19 KO mice reconstituted with WT, CXCR5 KO or CR1/2 B cells. Data in a-b show one out of 3 independently experiments performed. (d) Spleen sections from CD19 KO and reconstituted mice, 16h after injection of Phycoerythrin immune complex (PE-ICs). Sections were stained for CD169 to detect metallophilic macrophages (green) and CD35 to detect FDCs (blue). PE-ICs are in red. Sections from at least 2 mice per group were analysed in each experiment. (e) Spleen sections from WT, CD19 KO and CD19 KO mice that have been reconstituted with MZ B cells were stained with anti-SIGNR1 (red, top panel). Surfaces (blue) surrounding SIGN-R1 positive cells (SIGN-R1-surface) were created using the Imaris software. SIGN-R1-surfaces overlaying SIGN-R1+ cells are shown in the lower panel across the entire field of view. On the right, quantification of the frequencies of SIGN-R1+ cells, calculated as the sum of SIGN-R1-surface areas (micro-m^2) normalized to the total surface area of the section (micro-m^2). Each circle represents data from one analyzed section.

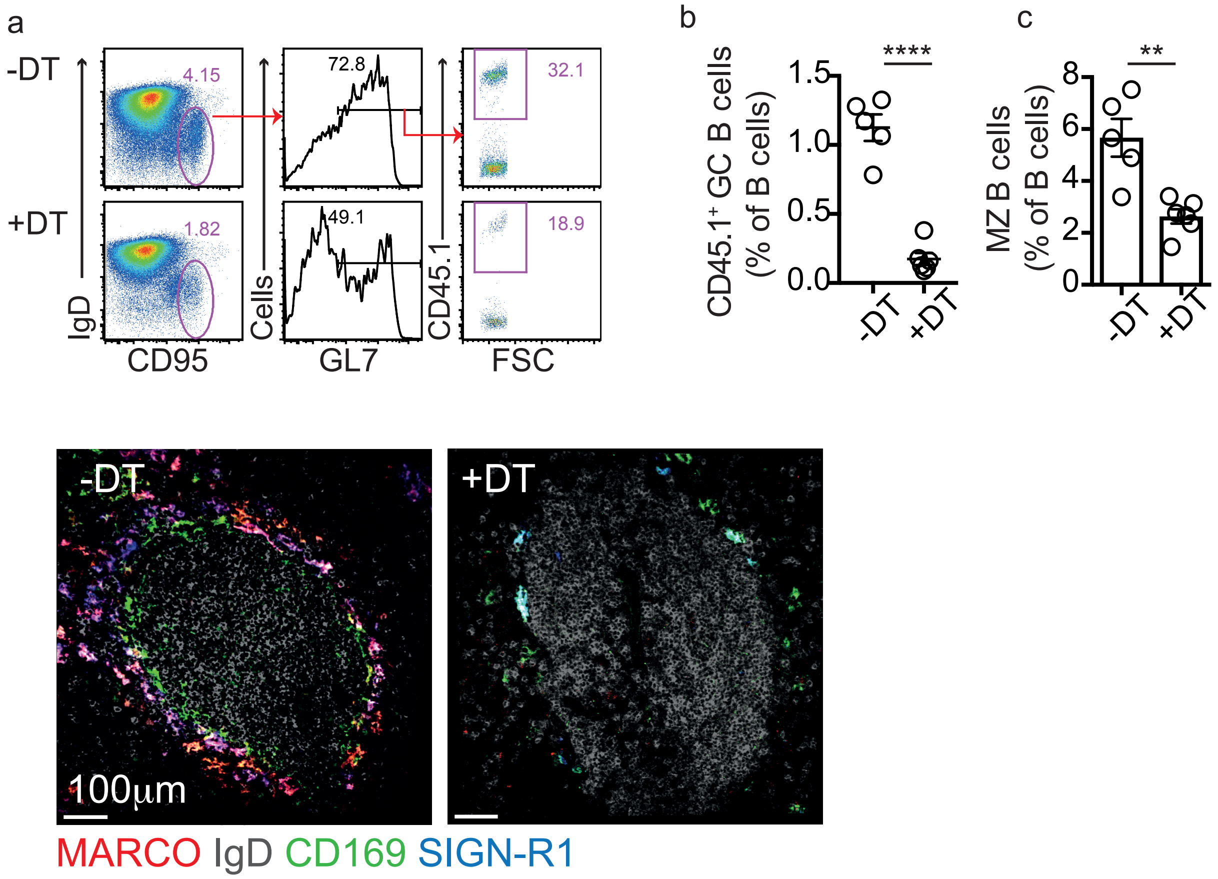

Figure S2. Depletion of MZ macrophages impairs GC B cell responses

CD169-DTR mice were treated with or without DT twice (days -5 and -2) prior to co-transfer of OTII T cells and $CD45.1^{+}$ MD4 B cells. The next day, mice were immunized with HEL-OVA (i.p). Spleens were analysed 8 days later. (a) Top, flow cytometry plots showing gating strategy to determine the frequencies $CD45.1^{+}$ MD4 B cells within the GC compartment. Bottom, immunofluorescence analysis of spleen sections from DT treated and untreated mice stained for CD169 (green), SIGN-R1 (blue) IgD (grey) and MARCO (red). Sections from at least 3 mice per group were analysed in each experiment. (b) Frequencies of $CD45.1^{+}$ GC B cells in the follicular compartment. (c) Frequencies of MZB cells (defined as live, $B220^{+}CD21^{high}CD23^{low}$ cells). Data represent one out of 4 independent experiments performed.

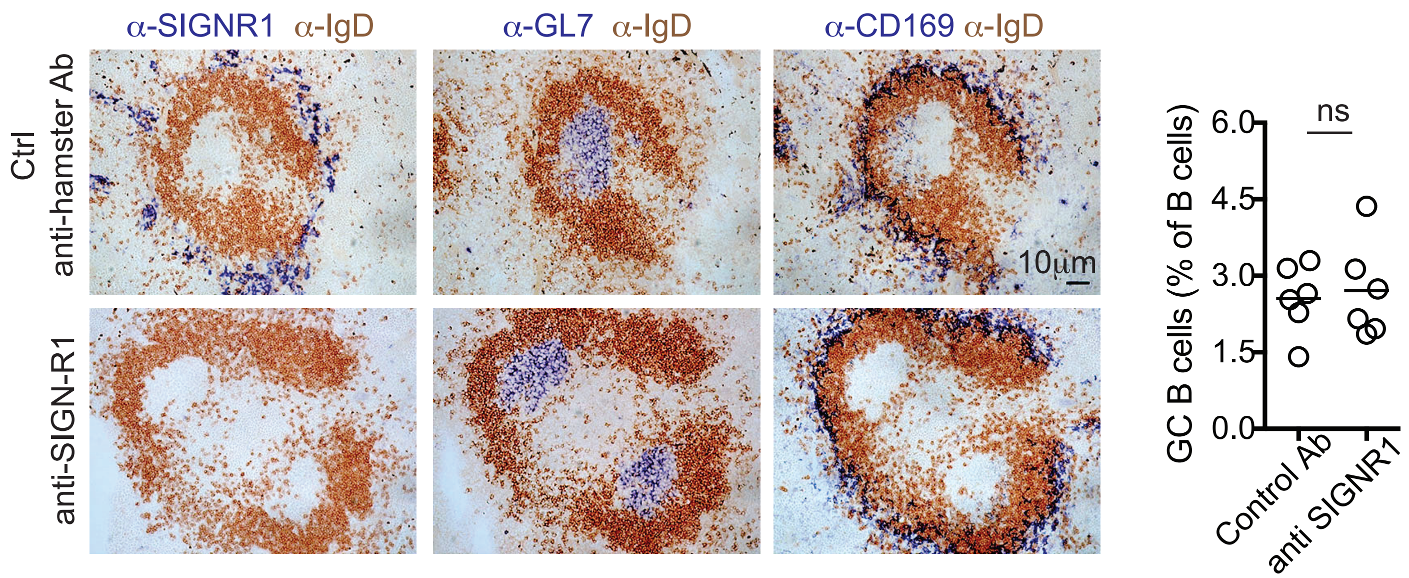

Figure S3. Effect of SIGN-R1 blockade on GC B cell responses.
Mice were injected intravenously with control anti hamster (Ctrl, upper panel) or hamster anti-SIGN-R1 (22D1, lower panel) antibody. One day later, mice were injected with SRBCs. Left, immunohistochemistry of consecutive sections from immunized mice stained for IgD (brown) and SIGN-R1, GL7 or CD169 (blue), as indicated. Sections from 3 (Ctrl) and 4 (22D1) treated mice were analysed in each experiment. Right, frequencies of GC B cells in the follicular compartment (defined as $B220^{+}IgD^{low}FAS^{+}GL7^{+}$ live cells). Figure shows one representative example out of 3 independent experiments.

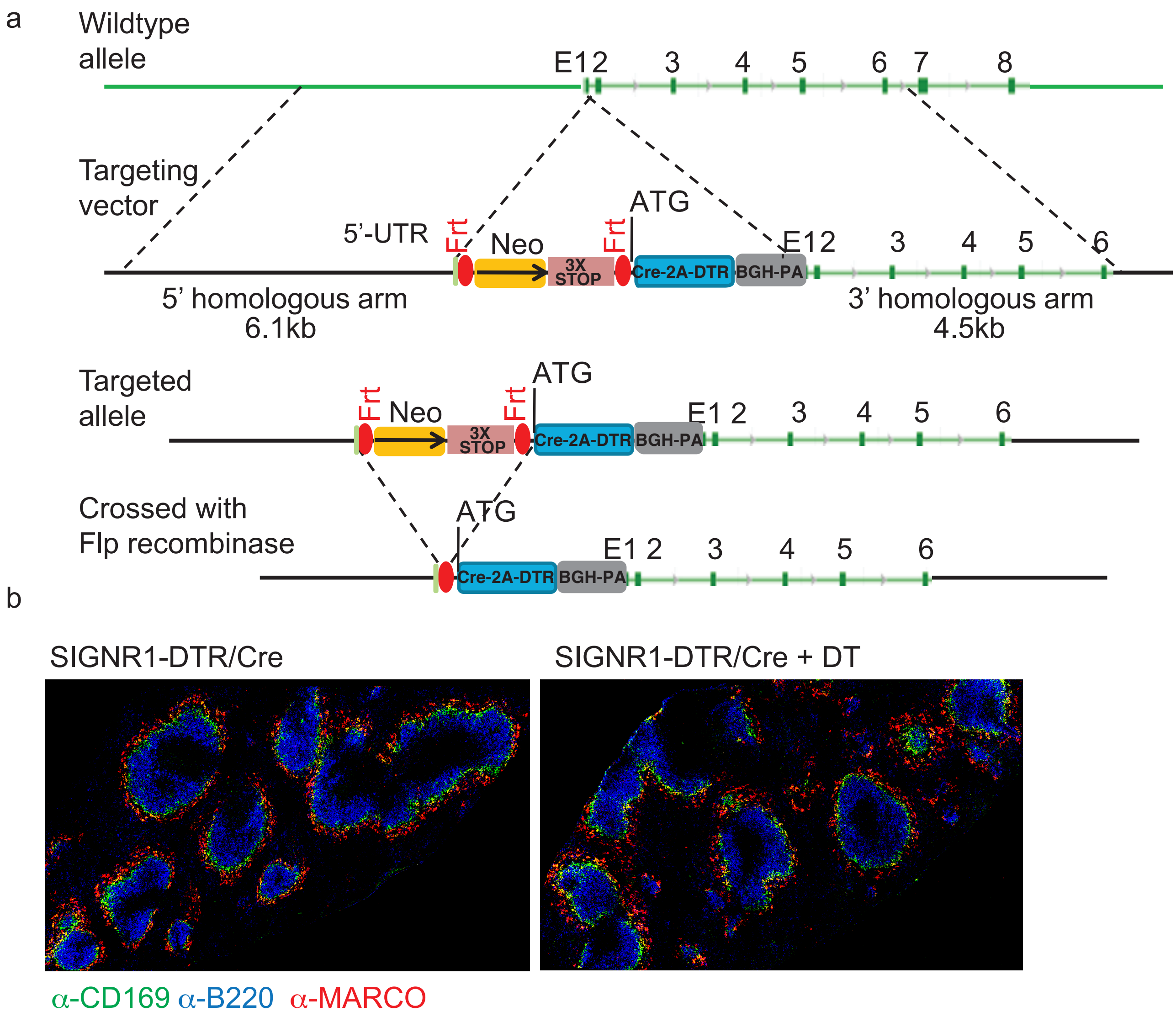

Figure S4. Generation of SIGN-R1-Cre/DTR mice

(a) Schematic of the targeting strategy. From top to bottom, the structures are shown for the WT allele, the targeting vector, the targeted allele, and the targeted allele after being crossed to a Flp-recombinase mouse. Exons are shown as boxes (E1-8). A Frt-flanked neomycin (neo) cassette followed by a STOP cassette containing 3 stop sequences (X3 STOP) and the expression cassette containing the cDNA for Cre recombinase (iCre) and the human diphtheria toxin receptor (DTR) separated by a 2A sequence and followed by the bovine growth hormone polyadenylation signal (BGH-PA) are indicated. The homology arms spanning the 5' and 3' UTR are shown.

(b) Immunofluorescence analysis of spleen sections from DT treated or untreated SIGN-R1-Cre/DTR+/- mice. Sections were stained for CD169 (blue), MARCO (red) and B220 (blue).

Images represent one out of at least 3 mice tested.

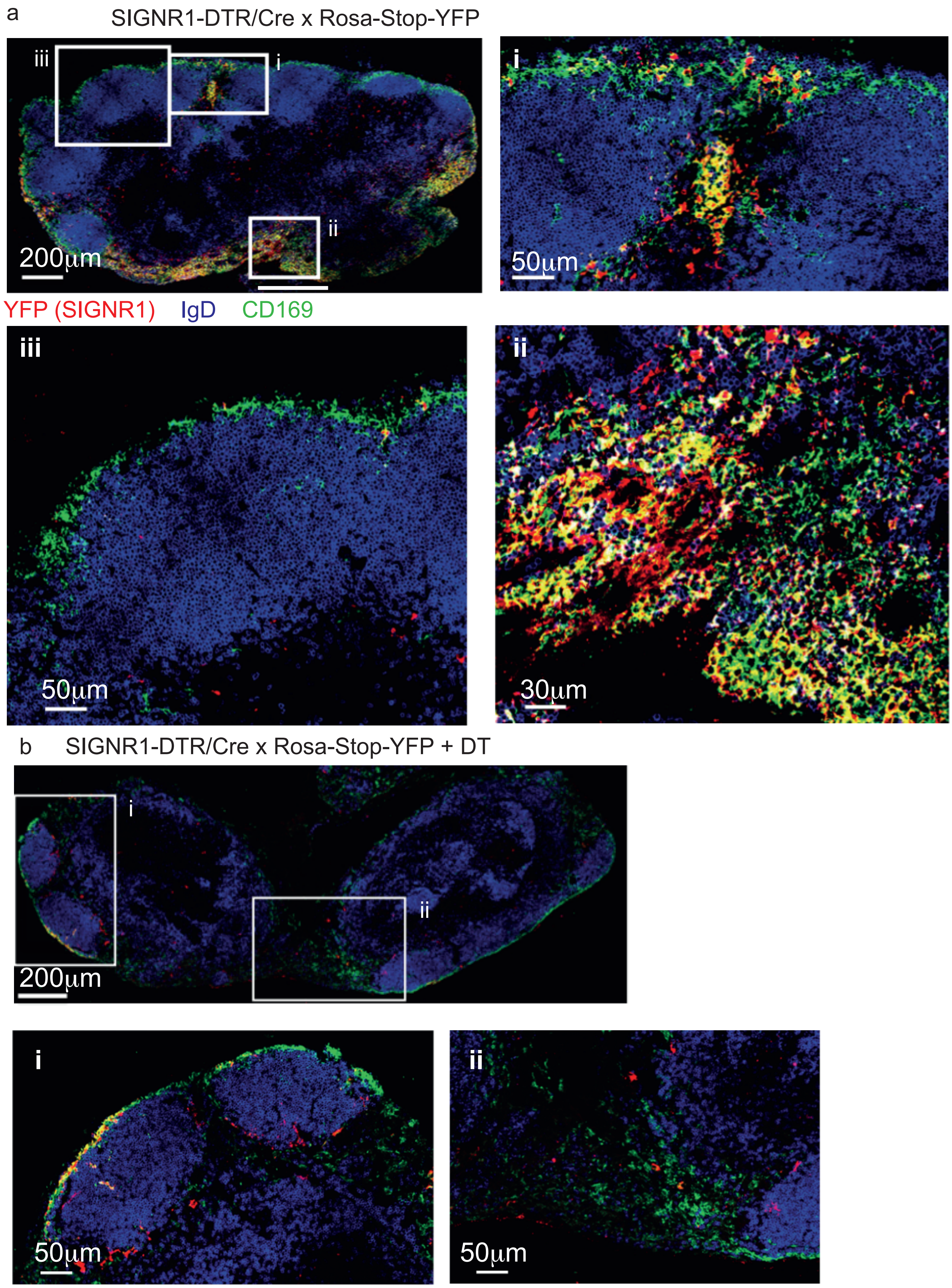

Figure S5. Selective ablation of SIGN-R1+ cells in lymph nodes of SIGN-R1-Cre/DTR mice
Immunofluorescence analysis of spleen sections of SIGN-R1-Cre/DTR+/- x R26-stop-YFP mice before (a) and after (b) DT treatment. Sections were stained for CD169 (green) and IgD (blue). Red, YFP signal. Boxed regions in top left images are shown in enlargements. Images represent one out of at least 3 mice tested.

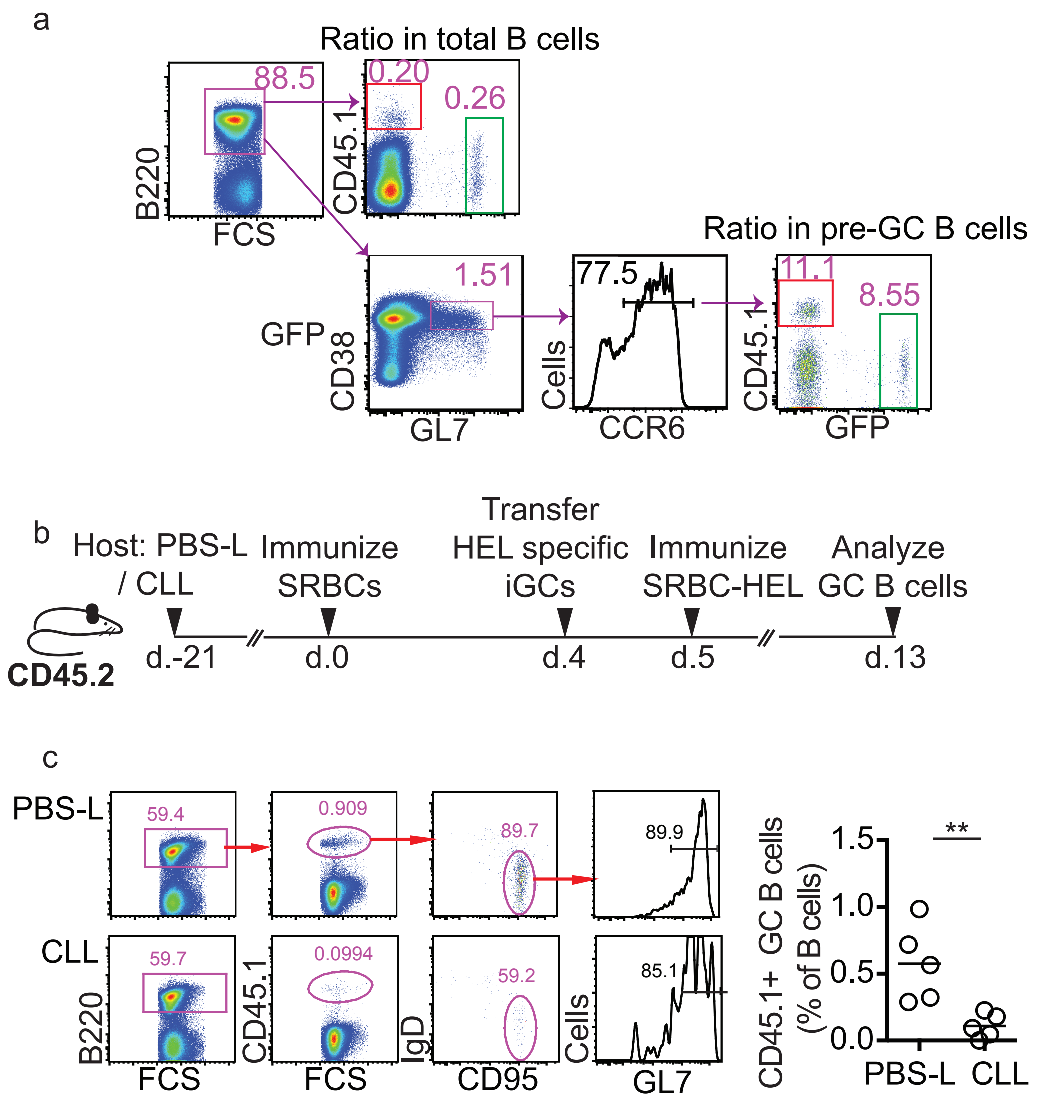

Figure S7. Transfer of pre-GC B cells from SIGN-R1 macrophage sufficient and depleted mice

(a) Figure corresponds to data shown in Fig 4c-d. Flow cytometry plots showing gating strategy to determine the ratio between B cells derived from PBS-L ($CD45.1^+$) and CLL (GFP^+) treated donors before transfer to SRBC-immunized mice. Top panel shows ratio of CD45.1/GFP cells in the total B cell compartment. Lower panel shows the ratio in the pre-GC B cell compartment, gated as shown. (b,c) Ex-vivo 'induced GC B cells' (iGCBs) were generated from MD4 B cells in vitro. iGCBs were transferred into mice that were treated with CLL or PBS-L 3 weeks earlier and that have been immunized with SRBCs 4 days prior to iGCB cell transfer. One day after transfer, mice were immunized with SRBC-HEL followed by flow cytometry analysis at day 8. (b) Graphic representation of the experimental setup. (c) Percentage of $CD45.1^+$ GC B cells within the follicular compartment recovered from recipients.

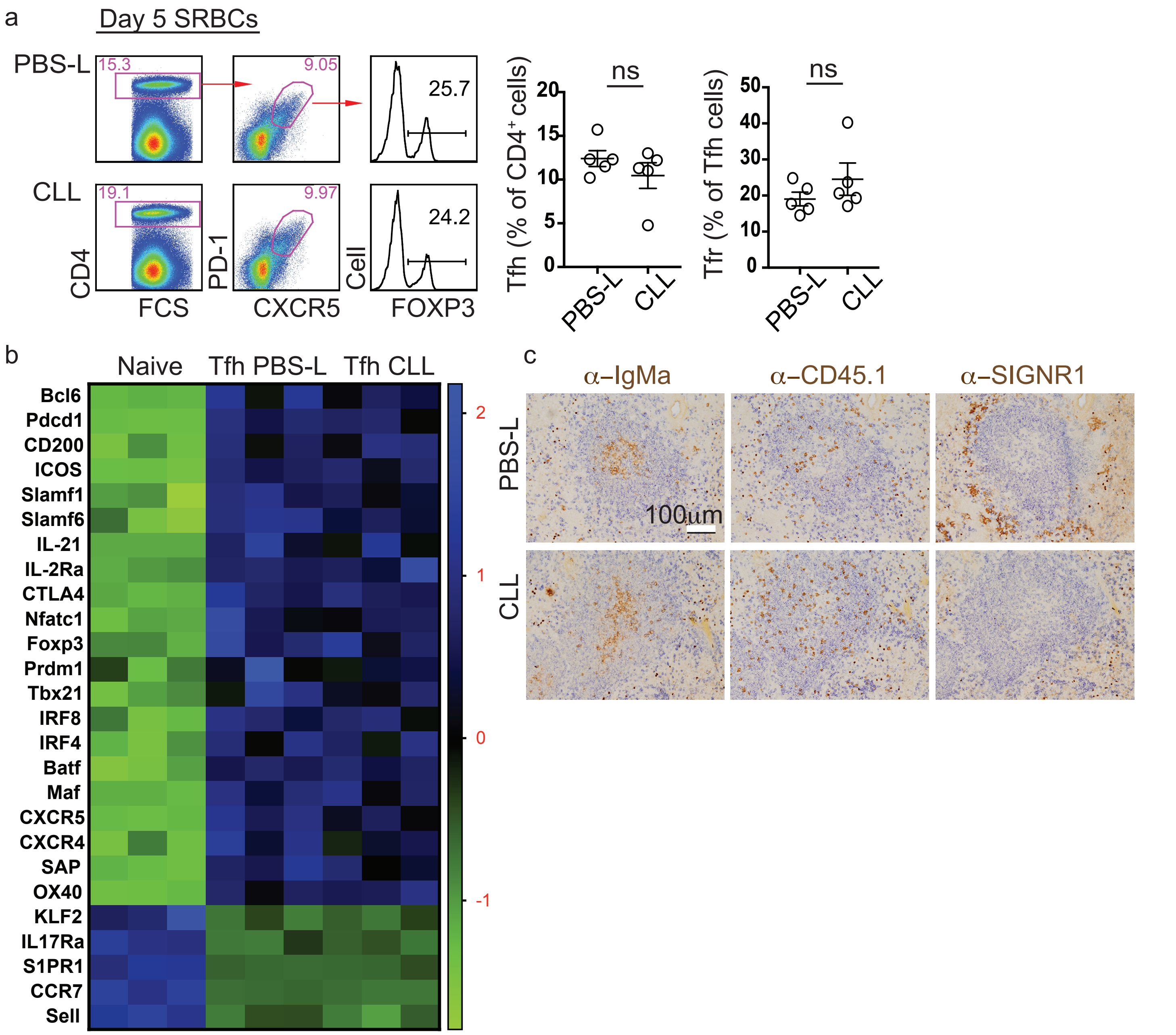

Figure S8. Tfh and Tfr differentiation in SIGN-R1 macrophage-depleted mice

(a) Frequencies of Tfh and Tfr in SIGN-R1 macrophage-depleted mice. Mice were treated with PBS-L or CLL 3-4 weeks prior to immunization with SRBCs. Mice were analysed 5 days post immunization. Left, FACS plots showing gating strategy to determine frequencies of Tfh and Tfr cells. Plots were pre-gated on B220$^-$ live cells. Right, percentage of Tfh and Tfr cells. Data represents one out of 3 experiments performed. (b) RNA-seq analysis of Tfh cells (B220$^-$ CD4$^+$ PD-1^{high} CXCR5^{high} l ive cells) and naïve T cells (B220$^-$ CD4$^+$ live cells) sorted 3.5 days post immunization of mice that were pre-treated with PBS-L or CLL 3 weeks earlier. Heat map shows expression of Tfh signature genes in naïve CD4$^+$ T cells compared to Tfh cells from CLL or PBS-L treated mice. (c) Positioning of Tfh cells in the follicles of SIGN-R1 macrophages depleted mice. CD45.2$^+$ IgMa MD4 and purified CD45.1$^+$ OTII cells were co-transferred into CD45.2$^+$ recipient mice that were treated with PBS-L or CLL 3 weeks earlier. The next day, mice were immunized with HEL-OVA. Five days post immunization, spleens were frozen and consecutive sections were stained for IgD (blue) and IgMa (to identify transferred MD4 B cells, brown), CD45.1 (to identify transferred OTII cells, brown) or SIGN-R1 (brown). Shown one representative example out of 2 experiments performed. Sections from spleens of at least 3 mice were analysed in each experiment.
